# Editorial: Multiple Identities Management: Effects on (of) Identification, Attitudes, Behavior and Well-Being

**DOI:** 10.3389/fpsyg.2017.02258

**Published:** 2017-12-22

**Authors:** Clara Kulich, Soledad de Lemus, Natasza Kosakowska-Berezecka, Fabio Lorenzi-Cioldi

**Affiliations:** ^1^Social Psychology, Section of Psychology, Faculty of Psychology and Educational Sciences, University of Geneva, Geneva, Switzerland; ^2^Department of Psychology, Centro de Investigación Mente, Cerebro y Comportamiento, University of Granada, Granada, Spain; ^3^Division of Cross-Cultural Psychology and Psychology of Gender, Institute of Psychology, Faculty of Social Sciences, University of Gdansk, Gdansk, Poland

**Keywords:** multiple identities, social identification, dissonance, coping, conflict, well-being

Individuals belong to gender, ethnic, or national groups; they can be categorized depending on their religious beliefs, or the activities they are involved in such as professions, political groups, etc. These multiple social identities differ considerably in the way they are acquired (e.g., inherited or achieved through accomplishments), in their relative stability or malleability, and in the value which they assign to the individual (e.g., low vs. high social status).

Inherited identities (based on gender, skin color, class background, etc.), and in some cases achieved ones (e.g., through migration or professional mobility), cannot be voluntarily dismissed. Although inherited and achieved social identities tend to correspond in their value and content over time (Bourdieu, 1979; Ridgeway and Erickson, 2000), identities can differ in status and value, and create distressing experiences which call for a coping strategy in order to increase identity fit (e.g., Deaux and Greenwood, 2013; Turner-Zwinkels et al., 2015). However, there is also evidence that multiple identities may provide a pathway to gain social support and positively influence individuals' well-being (Walter et al., 2015).

When individuals face conflicting identities in terms of status or value, they use different coping strategies. Individuals may attempt to discard one of the identities, use in turn one or the other, or integrate or fuse both identities (e.g., Roccas and Brewer, 2002; Deaux, 2008; Shields, 2008; Berry and Sabatier, 2011). Identities may also change or be newly developed in particular contexts (e.g., politicized, opinion-based, or solidarity-based groups; McGarty et al., 2009). The type of strategy is also likely to have an impact on well-being, as it has the potential to reduce dissonance and distress (e.g., Sampson, 1969; Jetten et al., 2012). Research shows that upward (Derks et al., 2011; Kulich et al., 2015) and downward (Jetten et al., 2015) individual mobility impinge on attitudes and support for other ingroup members. Moreover, conditions such as situational threats to a social identity (e.g., Kosakowska-Berezecka et al., 2016), as well as the type of integration culture (e.g., colorblindness vs. multiculturalism) moderate whether multiple identities produce positive or negative outcomes (Wilton et al., 2015).

In this Research Topic, we focus on contexts in which two or more social categories are simultaneously salient. Eighteen papers present empirical research, as well as novel theoretical considerations, to understand how such multiple identities are being managed by the individuals holding them. We were particularly interested in the positive and negative outcomes they bring for individuals' well-being, and in the importance of multiple group memberships for intergroup relations. In the following, we provide a brief discussion of, and structure for, the main themes included in this Research Topic.

## Multiple identity configurations—benefit or cost?

Identity configurations take many different forms depending on contextual factors. Looking at the antecedents of different identity configurations, Repke and Benet-Martínez highlight that the structure of an immigrant's social network and interconnection of same ethnicity (rather than the number of co-ethnic and host individuals in the networks) play a significant role in predicting whether individuals develop coexisting cultural identifications, conflicting cultural identifications, or a mixture of the two.

Belonging to a number of groups may be beneficial, as group membership enhances individuals' well-being and other positive outcomes. Zhang et al. show across diverse groups of bicultural Canadians that having an integrated bicultural identity is related to being more consistent across roles and more congruent and less ambiguous about self-evaluations. Thus, both content of heritage culture and the dynamic process of integrating cultural identities affect self-consistency among biculturals. Levels of identification with multiple groups affect the group members' performances or preferences. Leicht et al. show that women are more likely to manifest leadership aspirations in a work scenario when they indicate simultaneous high identifications with women and with feminism, but only in a context where gender counter-stereotypes are made salient (as compared to a stereotypical context). Moreover, cross-sectional work by Steffens et al. suggests that multiple social identities are positively related with better health and increased well-being in retirement because they allow individuals to both give and receive social support. However, these positive outcomes are often contingent on specific conditions. In this way, Chang et al.'s studies and meta-analysis propose that, although for Europeans multiple group memberships have a number of positive outcomes, for Asians they do not convey better well-being because receiving social support is perceived as burdening others. It is thus uncomfortable for Asians to draw from such psychological resources. In accordance, Sønderlund et al. argue, based on correlational studies, that specific features of the groups, such as their social value and visibility to others, need to be considered beyond the number of identities. Indeed, low value of highly visible identities makes them more of a burden than a benefit for individuals' well-being.

Multiple group memberships may further engender dissonance and threat and thus be troubling for the individual. Identities that differ in value and content are particularly common among members of inherited low status groups in work situations. For instance, Veldman et al. find that female police officers' experience of being gender-dissimilar from the work-team members is associated with gender-work identity conflict. As a consequence, female police officers identify less with their team, leading to negative work and health outcomes such as lower work satisfaction and motivation, higher burnout, and turnover intentions. von Hippel et al. further show that the incompatibility of female gender and work identities impacts on attitudes toward family-friendly policies. They demonstrate that women suffering from stereotype threat perceive such policies as having negative career consequences—although paradoxically they are still more inclined to use them.

## How to solve multiple identity conflict

When identity conflict occurs, different identity strategies serve to reduce this inconsistency. Jones and Hynie focus on the experience and management of conflict between different types of multiple identities: roles (e.g., being a student), relational (being a friend), or social identities (nationality). They suggest four strategies of identity management: reconciliation, retreat, realignment, and reflection.

Matschke and Fehr analyze the impact of incompatibilities between an established social identity and a potential new social identity in an acculturation context. They suggest that lack of compatibility between cultural identities leads to higher disidentification with the receiving society. Depending on cultural self-construal (individualistic or collectivistic), individuals make use of different resources (related to either intrinsic or extrinsic motivation) to fight the negative effect of incompatibility on the social identity.

And finally, Meeussen et al. focus on managing potentially conflicting work-family identities. They point to the importance of perceived gender norms in achieving compatibility between gender, family, and work. Their findings show that gender identity influences women's and men's work-family conflict resolution aspirations through social norms. Thus, changing these norms could allow both men and women to combine their multiple identities in a more effective and self-enhancing way.

## Multiple identities, intragroup, and intergroup relations

Multiple identities can compromise interpersonal relations by creating conflict within the group. In this line, Sankaran et al. present an experimental study in the context of the Indian caste system, where social mobility is strongly restricted. They find evidence for the *black sheep effect*: When an ingroup member (as compared to an outgroup member) threatens the high-castes' social identity by violating an ingroup norm (morality), high caste identity increases in concert with the derogation of the ingroup perpetrator. In parallel, Chipeaux et al. investigate, with correlational data, moves from a disadvantaged ingroup to a higher status outgroup. They observe a decrease in ingroup concern among mobile individuals and among those who anticipate moving up the hierarchy, compared to non-mobile individuals. These results suggest that multiple identities with differing statuses lead to identity conflict, resulting in the discount of the low-status ingroup. Finally, Chiou and Mercado analyze whether biculturals have fixed or shifting loyalties toward their home/host culture. Their findings reveal that the degree to which biculturals manifest loyalty to each of the respective cultures is easily influenced by a priming procedure, demonstrating that bicultural loyalties are rather malleable.

Further, multiple identities can contribute to a more nuanced understanding of intergroup relationships. For instance, van Breen et al. show that a multiple identity approach to gender allows predicting women's attitudes toward gender stereotypes as well as collective action. They propose that women's attitudes are regulated by two dimensions of gender identity: identification with women and with feminists (politicized identity).

Indeed, interacting social identities play an important role in mobilizing resources for activism. Pereira et al. analyze the “sedating” effect of positive intergroup contact for Roma minority activism. They demonstrate that positive contact reduces Roma's ethnic identification and activism but only among low national identifiers. Thus, national identification can *buffer* against negative effects of contact on collective action. Similarly, in the context of Ukraine ban protests of 2014, Chayinska et al. show that national and politicized identities (online and street protest groups) predict support for collective action if protest is perceived as legitimate and politicized identities as compatible.

Finally, Levy et al. focus, in a theoretical contribution, on how the salience of multiple group memberships impacts on within-group and between-groups relations. They define *gateway groups* as individuals who simultaneously belong to two groups who are in conflict and propose that such gateway groups operate as both bridges and barriers in intergroup relations, thus improving or worsening intergroup relations. They conclude that the way in which gateway groups build their multiple identities and shift across categories play an important role in reducing group stereotypes and fostering tolerance toward outgroups.

## Conclusions

The aim of this Research Topic was to extend a growing body of research on multiple identities by focusing on how people negotiate conflicting social identities and the consequences of such negotiations. The present collection of research findings investigated the moderating role of group status, group visibility, dissimilarity from others, diversity cultures, and types of identity configurations on the ways individuals handle their multiple identities. They further investigated the particularities of psychological constructs such as social identification, ingroup and inter-group attitudes and behaviors (e.g., collective action and social mobility), and well-being in the context of multiple identity configurations of different kinds (e.g., gender, professional, and politicized identities). Future research should focus on a systematic analysis which extends the correlational level in order to better understand the specific contexts and the underlying processes that determine whether multiple identities are beneficial or detrimental for the individual's well-being. Moreover, research should examine how social perceptions of multiple identities impact the process of coping and identity management in individuals who are holding these identities. This implies bringing together the literature on the perspective of individuals holding multiple identities, presented here, and the literature on attitudes toward individuals with multiple identities.

## Author contributions

CK, SdL, NK-B, and FL-C all drafted the Research Topic proposal, took on editorial tasks, and participated in writing and commenting the editorial.

### Conflict of interest statement

The authors declare that the research was conducted in the absence of any commercial or financial relationships that could be construed as a potential conflict of interest.
